# DNdisorder: predicting protein disorder using boosting and deep networks

**DOI:** 10.1186/1471-2105-14-88

**Published:** 2013-03-06

**Authors:** Jesse Eickholt, Jianlin Cheng

**Affiliations:** 1 Department of Computer Science, University of Missouri, Columbia, MO 65211, USA; 2 Informatics Institute, University of Missouri, Columbia, MO 65211, USA; 3 C. Bond Life Science Center, University of Missouri, Columbia, MO 65211, USA

**Keywords:** Protein disorder prediction, Disordered regions, Deep networks, Deep learning

## Abstract

**Background:**

A number of proteins contain regions which do not adopt a stable tertiary structure in their native state. Such regions known as disordered regions have been shown to participate in many vital cell functions and are increasingly being examined as drug targets.

**Results:**

This work presents a new sequence based approach for the prediction of protein disorder. The method uses boosted ensembles of deep networks to make predictions and participated in the CASP10 experiment. In a 10 fold cross validation procedure on a dataset of 723 proteins, the method achieved an average balanced accuracy of 0.82 and an area under the ROC curve of 0.90. These results are achieved in part by a boosting procedure which is able to steadily increase balanced accuracy and the area under the ROC curve over several rounds. The method also compared competitively when evaluated against a number of state-of-the-art disorder predictors on CASP9 and CASP10 benchmark datasets.

**Conclusions:**

DNdisorder is available as a web service at http://iris.rnet.missouri.edu/dndisorder/.

## Background

Many proteins contain regions which do not adopt a stable tertiary structure in their native state. These regions have been identified by various terminologies in the literature and names include disorder regions [1], intrinsic disorder [2], intrinsically disordered regions (IDRs) [3] and intrinsically unstructured proteins (IUPs) [4]. This disorder or lack of structure may be limited to a particular region or regions of a protein chain or may extend throughout the entire protein. Disorder can also be transitory in nature and linked to a certain state of a protein such as bound or unbound (e.g., a region may be disordered when the protein is unbound but then fold into a stable structure upon binding to a ligand).

Protein disordered regions are of particular interest due to their involvement in signalling pathways, transcription and translation [4,5]. Their inherit flexibility make it possible for a protein to bind to many partners and make them attractive targets for drug development. Several methodologies have been proposed for disorder-based rational drug design (DBRDD) and some peptides have already been designed which block interactions between structured and unstructured partners [6,7]. As a result, methods are needed to accurately predict protein disorder and aid in the search for new drug targets.

Recent estimates indicate that there are over 60 protein disorder predictors [8]. A number of comprehensive reviews on disorder predictors exist, outlining methodology and availability [2,9]. Generally speaking, existing methods for the prediction of protein disorder can be coarsely categorized as propensity-based, machine learning based, contact-based or a meta-method [8]. Propensity-based predictors work on the premise that certain types of amino acid residues are more likely to be found in the core of an ordered region than a disordered region. Likewise, there are particular residues which appear to be over represented in disordered regions. A statistical analysis of known ordered and disordered proteins allows for the creation of disorder propensities which can be used to predict disorder [10-13]. This approach is fast and simple but does not make use of the data in an optimized way. Predictors based on machine learning, such as neural networks [14] or support vector machines [15,16], also make use of experimental data on ordered and disordered residues but do so via sophisticated learning algorithms which allow for more than sequence data as input. High dimensional functions are fit to the input features through training and then used to predict residue disorder. This does allow for optimized use of the experimental data but results in a prediction approach that is based on a complex function. It is often difficult to understand how the function depends on its input and this approach lacks an intuitive rationale as to how the prediction is made. Methods based on residue-residue contacts attempt to determine if sufficient interactions take place to pull the protein chain into a stable conformation. Residue-residue contact data may come in the form of predicted packing density or predicted residue-residue contacts [17,18]. Meta predictors, or meta methods, are combinations of the aforementioned methods and are constructed by combining several predictors. This can be done by a simple averaging of the output from each method or in a performance weighted manner. This usually results in a slight improvement in performance [1,19] but the approach may not be practical on a genomic scale if it depends on too many disorder predictors.

Here we present a new sequenced-based predictor of protein disorder using boosted ensembles of deep networks (DNdisorder). To the best of our knowledge this is the first use of deep networks for disorder prediction. By using CUDA and graphical processing units we were able to create very large, deep networks to predict disordered regions. We also combined this novel approach with another sequence based disorder predictor to create a small, meta predictor. The meta predictor provides a boost in performance with a negligible increase in prediction time. To evaluate our methods, we compared them to a number of other disorder predictors on a common benchmark dataset as well as in the recent round of the Critical Assessment of Techniques for Protein Structure Prediction (CASP10) experiment. The results of this evaluation show that our novel approach compares competitively with many state-of the-art disorder predictors. This indicates that boosted ensembles of deep networks can be used to predict protein disorder regions.

## Methods

### Datasets

**Figure 1:**
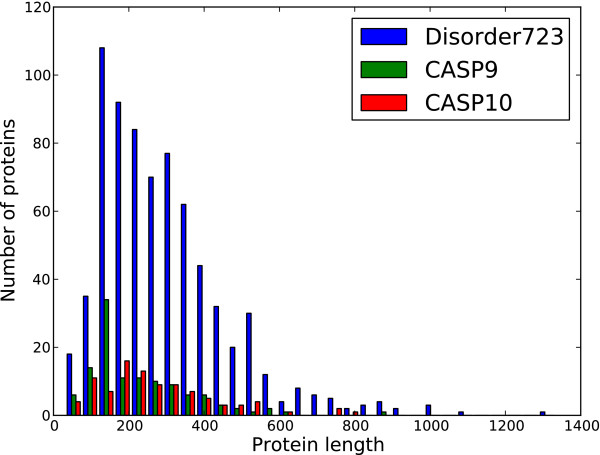
**Distribution of protein lengths for DISORDER723, CASP9 and CASP10 datasets.** The distribution of the length of the proteins that make up the DISORDER723, CASP9 and CASP10 dataset. These datasets consist of 723, 117 and 95 proteins respectively.

**Figure 2:**
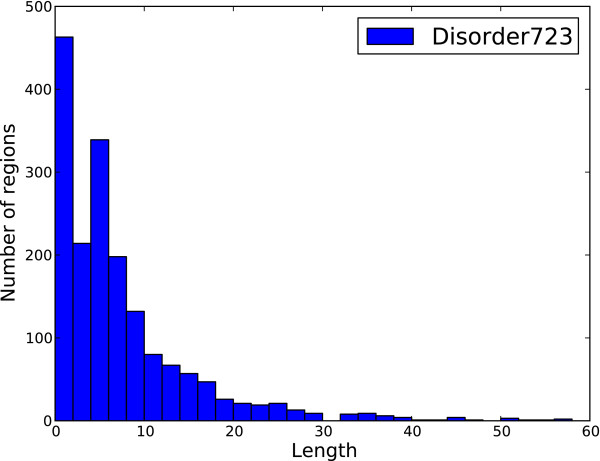
**Distribution of disordered region length for DISORDER723.** The distribution of the length of disordered regions for the training dataset DISORDER723. Not included in this figure are 19 regions with lengths longer than 60 residues.

**Figure 3:**
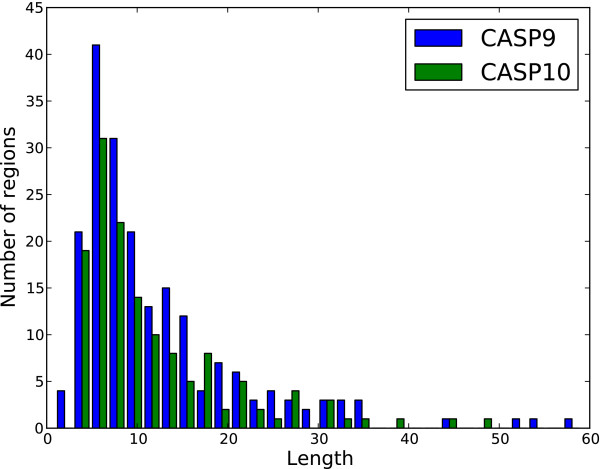
**Distribution of disordered region length for CASP9 and CASP10 datasets.** The distribution of the length of disordered regions for the CASP9 and CASP10 evaluation datasets. Residues marked as ‘X’ by the CASP assessors were not considered as disordered when calculating this distribution.

The principle dataset used for training was DISORDER723, a set of 723 proteins originally constructed for the development of DISpro [20,21] and later PreDisorder [22]. It consists of proteins which are more than 30 residues in length and contain at least one disordered region 3 residues or longer in length. It is comprised of 13909 disordered residues and 201703 ordered residues (i.e., ~6.5% disordered). Additional datasets used for evaluation include CASP9 and CASP10, respectively comprised of 117 and 95 proteins and used during the CASP9 and CASP10 competitions. The CASP9 dataset consists of 23656 ordered residues and 2427 disordered residues (i.e., ~9.3% disordered) and the CASP10 dataset contains 22673 ordered residues and 1597 disordered residues (i.e., ~6.6% disordered). All the accession dates for the proteins in DISORDER723 predate March 2003 and well before the CASP9 and CASP10 competitions which took place during the years of 2010 and 2012. The distribution of the lengths of the proteins comprising these datasets is shown in Figure 1. Figures 2 and 3 represent the distribution of the lengths of the disorder regions in the datasets. For the CASP9 and CASP10 datasets, the protein sequences and experimentally determined order/disorder state were obtained from the official CASP website [23]. Residues that did not receive a disorder/order classification by the CASP assessors (i.e., those designated as ‘X’) where not considered to be disordered in our assessment. The dataset DISORDER723 is available also online and available for download [24].

### Restricted Boltzmann machine and deep networks

Conceptually deep networks (DNs) are similar to neural networks but contain more layers and trained in a slightly different manner. One way to train DNs is using a layer by layer unsupervised approach. Here, the idea is to first learn a good model or representation of the data irrespective of the label of each data point. This process allows one to first learn relationships that might exist in data. After theses relationships are learned, a supervised learning technique such as a 1 layer neural network can be trained on the learned, higher level representation of the data. Intuitively the general idea behind such an approach is that to do effective classification it is useful to first learn the structure (i.e., features) of the data. Relatively recent developments in training algorithms for DNs has lead to their successful use in a number of areas such as image recognition [25], speech recognition [26], text classification and retrieval [27] and residue-residue contact prediction [28]. There are a number of introductions and overviews to deep learning and deep networks in the literature including two foundational works by Hinton *et al*. [29,30] and an overview of training deep networks [31].

The general framework used for our disorder predictor was a collection of boosted ensembles comprised of deep networks. Each DN is a deep, multilayer neural network that is trained layer by layer using restricted Boltzmann machines and then fine tuned using a back propagation procedure. A restricted Boltzmann machine (RBM) is a two layer network with one layer termed the visible layer which takes on the values to be modeled and the other is the hidden, or latent, layer [32,33]. In its purest form, the nodes in a RBM are stochastic and binary. Symmetric, weighted connections exist from every node in the visible layer to every node in the hidden layer. There are no connections within a layer and every node has a bias. In this context, the energy of a particular configuration can be defined as

$$E\left(v,h\right)=-\sum_{i}b_{i}v_{i}-\sum_{j}c_{j}h_{j}\sum_{i,j}h_{j}v_{i}w_{\mathrm{ij}}$$

where *h*_*j*_ and *v*_*i*_ are the states of the j^th^ hidden and i^th^ visible nodes, *c*_*j*_ and *b*_*i*_ are biases for the j^th^ hidden node and i^th^ visible node, respectively. *w*_*ij*_ is the weight of the symmetric connection between the i^th^ and j^th^ nodes. By summing over all possible configurations of h and normalizing (*Z*), it is possible to define a probability for a particular configuration of visible nodes, *v*.

$$\text{p}\left(\text{v}\right)=\sum_{\text{h}}\frac{\text{e}-\text{E}\left(\text{v},\text{h}\right)}{\text{Z}}$$

Training a RBM entails adjustments in the weights and biases such that the probability assigned to training data is higher than randomly chosen configurations of the visible nodes. This is typically done using a process known as contrastive divergence [32]. In this work, the weights in the n^th^ round of training were updated using the following rules:

$$\begin{array}{l} \Delta^{\left(n\right)}w_{\mathrm{ij}}=\varepsilon \left\{\left(<v_{i}p_{j}{>}_{\mathrm{data}}-<p_{i}^{\left(1\right)}p_{j}^{\left(1\right)}{>}_{\mathrm{recon}}\right)-\eta \;w_{\mathrm{ij}}\right\}\\ \Delta^{\left(n\right)}a_{i}=\varepsilon \left\{\left(<v_{i}{>}_{\mathrm{data}}-<p_{i}^{\left(1\right)}{>}_{\mathrm{recon}}\right)+v\;a_{i}^{\left(n-1\right)}\right\}\\ \Delta^{\left(n\right)}b_{j}=\varepsilon \left\{\left(<p_{j}^{\left(0\right)}{>}_{\mathrm{data}}-<p_{j}^{\left(1\right)}{>}_{\mathrm{recon}}\right)+v\;b_{j}^{\left(n-1\right)}\right\} \end{array}$$

More specifically, in these update rules the angle brackets represent averages which are taken over the batch. $p_{j}^{\left(0\right)}$ is the probability that the j^th^ hidden unit will be activated and can be calculated by applying the sigmoid function to the bias for the j^th^ hidden unit plus the sum of the products of each visible unit times the weight of the connection between the visible unit and the j^th^ hidden unit.

$$p_{j}^{\left(0\right)}=\sigma \left(\sum_{i}v_{i}w_{\mathrm{ij}}+b_{j}\right)$$

where the σ() represents the sigmoid function. $p_{i}^{\left(1\right)}$ is the probability that the i^th^ visible unit will be activated and calculated in a similar fashion to *p*^*(0)*^*.* In this case, the biases for the visible units are used as well as the states of the hidden units. The state of the j^th^ hidden unit is represented by h_j_ and set to 1 with probability $p_{j}^{\left(0\right)}$.

$$p_{i}^{\left(1\right)}=\sigma \left(\sum_{j}h_{j}w_{\mathrm{ij}}+a_{i}\right)$$

$p_{i}^{\left(1\right)}$ is the probability that the j^th^ hidden unit will be activated when driven by the probabilities of the reconstructed visible nodes (ie, *p*^*(1)*^). It is calculated in the same manner as $p_{j}^{\left(0\right)}$ but with $p_{i}^{\left(1\right)}$ used in place of *v*_*i*_. The update rules also contain three additional parameters which can be tuned for the particular application. These are the learning rate (ɛ), the weight cost (η) and momentum (υ). The values for these parameters and the update rules were selected based on recent findings describing how to train RBMs in practice [31]. In this work, the learning rate ɛ was set to 0.01 for *w* and 0.1 for the biases and the weight cost η was set to 0.0002. The momentum *υ* was initially set to 0.5 and after 5 epochs of training increased to 0.9. Training for a RBM took place over 20 epochs using batches of 100 training examples. We did not attempt to optimize these parameters and the evaluation data was not consulted during training.

A principle use of RBMs is as a means to initialize the weights in a DN. This is done by learning the weights at each level in a step-wise fashion. The first layer is trained using the training data and the aforementioned training procedure for a RBM. After the weights have been learned, the probabilities for activating the hidden nodes are calculated for every example in the training data. These activation probabilities are then used as the input to train another RBM. This procedure can be repeated several times to create several layers. The last layer is a single layer neural network trained using the target values and the last set of activation probabilities. Finally, all of the nodes can be treated as returning real-valued, deterministic probabilities and the entire deep network can be fine tuned using the back propagation algorithm [29,30].

To work with large models and datasets we implemented the training and prediction processes for the method using matrix operations. This allowed us to use CUDAMat [34], a python library which provides fast matrix calculations on CUDA-enabled GPUs. With this implementation we were able to train very large DNs (e.g., over 1 million parameters) in a timely manner (i.e., under 2 hours).

### Predicting disordered residues

To predict disordered residues, we trained a number of boosted ensembles of DNs. The input for each DN came primarily from a fixed length window centered on the residues to be classified. For each residue in the window, structure based and sequence based values as well as statistical characterizations were used as features (see “Features used and generation” for full details). The targets were the order/disorder states of the individual residues in a small window of 3, 5 or 7 residues in size. For the input window size, we used lengths of 20, 25 and 30. In total, there were 5 input-target window combinations. These were 20 to 3, 25 to 3, 25 to 5, 30 to 5 and 30 to 7. Depending on the size of the input window there were between 644 to 964 input features which resulted in the DN having an architecture of (644 to 964)-750-750-350-(3, 5 or 7). Each layer in the network was initialized using a RBM via the previously described process. The entire network was fine tuned using the back propagation algorithm to minimize the cross-entropy error. This was done over 25 epochs using batches of 1000 training examples.

In order to create boosted ensembles, we trained a series of DNs using a sample of 60,000 training examples which came from the entire pool of training data. The training examples came from the dataset DISORDER723 and consisted of all target windows and their corresponding input window. Initially, all of the training examples had an equal chance of being included in the training sample. After each round, the training pool was evaluated using the newly trained DN and the pool was reweighted based on the performance of the classifier. The probability of training examples which were at least partially misclassified was increased while the probability of selecting a properly classified example was decreased. This was done using a modified version of AdaBoost [35]. In particular, let *x*_*i*_ represent the i^th^ example in the training pool and *y*_*i*_*∈* {0, 1} be the classes of the i^th^ example (0 represents an ordered residue and 1 a disordered residue). Furthermore, let *W*_*t*_*(i)* be the probability of selecting the i^th^ example from the training pool in the t^th^ round of boosting and call the DN classifier trained in round *t* to be *m*_*t*_(●) which outputs a value between 0 and 1. Note that since the target has multiple values (ie, 3, 5 or 7), the probability of selecting the training example was increased in a manner proportional to the number of misclassified residues in the target window. Let *β* represent the number of target residues misclassified. Now, after each round of boosting, *W*_*t*_*(i)* is updated via ɛ_t_, α_t_ and h_t_(●) in the following manner.

$$\begin{array}{l} \;h_{t}\left(i\right)=\left\{ \begin{array}{l} 0\;if\;m_{t}\;(xi)\;<0.5\\ 1\;if\;m_{t}\;(xi)\;\geq 0.5 \end{array}\right.\\ \;\in_{t}=\sum_{h_{t}\left(x_{i}\right)\neq y_{i}}W_{t}\left(i\right)\\ \;\alpha_{t}=\frac{1}{2}\left(\frac{1-\in_{t}}{\in_{t}}\right)\\ W_{t+1}\left(i\right)=\frac{W_{t}\left(i\right)}{Z_{t}}*\left\{ \begin{array}{l} e^{-\alpha_{t}}\;if\;h_{t}\;\left(x_{i}\right)=y_{i}\\ e^{\beta *\alpha_{t}}\;if\;h_{t}\;\left(x_{i}\right)\neq y_{i} \end{array}\right. \end{array}$$

After the 35 rounds of boosting, the final output of the ensemble (represented by *H(x*_*i*_*)*) is a performance weighted average of all of the DNs. It is a value between 0 and 1 and for any input *x*_*i*_ calculated as follows:

$$H\;\left(x_{i}\right)=\frac{\Sigma_{\left(m_{t}\left(x_{i}\right)>0.5\right)}\;\alpha_{t}}{\Sigma_{t}\;\alpha_{t}}$$

A caveat of our boosting procedure is that after 7 rounds of boosting, all of the probabilities for the examples in the training pool were reinitialized to a uniform distribution. This was done as we saw that the weights of a few challenging training examples became too large and effectively dominated the selection process. This type of phenomena has been seen elsewhere and can lead to over fitting or poor performance [36]. Indeed, DNs trained of these types of training samples did not generalize well and effectively limited boosting to a small number of rounds (i.e., less than 10). Thus, by reinitializing the weights after 7 rounds we were able to create larger ensembles.

### DNdisorder

The final step in the construction of our DN based disorder prediction was to combine the results from the various boosted ensembles into one prediction. Each boosted ensemble consists of 35 predictors and there are 5 input-target window combinations (i.e., 20–3, 25–3, 25–5, 30–3 and 30–7). Thus, in all there are 175 predictors. The per residue prediction for each boosted ensemble is made using the aforementioned approach (i.e., a performance weighted average). The final prediction is a simple average of the values produced by each boosted ensemble. This final value is the output of our method which we call the DNdisorder predictor.

### Sequence based meta approach

In addition to DNdisorder, our DN based disorder predictor, we developed a small, sequence based meta predictor. This approach which we call PreDNdisorder is a simple average of the outputs from DNdisorder and PreDisorder. PreDisorder is another fast sequence based predictor of disorder regions we developed and build upon 1D recursive neural networks [22].

### Features used and generation

A number of sequence based features were used as input into our disorder predictor. These included values from a position specific scoring matrix (PSSM), predicted solvent accessibility and secondary structure, and a few statistical characterizations. The predicted values for both solvent accessibility and secondary structure were obtained using ACCpro and SSpro from the SCRATCH cluster of tools [37]. The PSSM was calculated using PSI-BLAST [38] for 3 iterations against a non-redundant version of the nr database filtered at 90% sequence similarity. For statistical characterizations of the amino acid residues we used the Acthley factors which are five numeral values which characterize an amino acid by secondary structure, polarity, volume, codon diversity and electrostatic charge [39]. Finally, note that all feature values were scaled to be in the interval from 0 to 1 in order to be compatible with the input layer of a RBM.

As previously mentioned, the input to a DN is a fix length window centered on the target window (ie, those residues to be classified). For each residue in the input window we used two binary inputs for solvent accessibility (buried: 01, exposed: 10), three binary inputs to encode for the secondary structure (coil: 001, beta: 010, alpha: 100), five inputs for the Acthley factors and from the PSSM we obtained 1 value for the information score of the residue and 20 inputs for the likelihoods of each amino acid type at the position. Note that as a window slides across the protein sequence, part of it may extend beyond the ends of the sequence. Thus, there is the need for an additional binary feature which encodes whether or not the position in the window is contained in the sequence boundaries and actually corresponds to a residue. If a window position does not correspond to an actual residue then all of the residue specific features for that position are set to 0. In addition to the residue specific inputs, we also used four, real value global features which were the percent of total residues predicted to be exposed, the percent of total residues predicted to be alpha helix, the percent of total residues predicted to be in a beta sheet and the relative position of the target residues (ie, middle of target window ÷ sequence length). Since three different sizes of input windows were used (ie, 20, 25 and 30) the total number of input features ranged from 644 to 772 to 964.

### Evaluation metrics

The output of DNdisorder is a real valued number from 0 to 1 with 0 corresponding to an ordered residue (0) and 1 a disordered residue (D). Given a set decision threshold residues can be classified as ordered if the output of DNdisorder is less than the decision threshold or as disordered if the output is greater than the threshold. After predictions are made it is possible to determine the number of true positives (TP), false positives (FP), true negatives (TN) and false negatives (FN). True positives are residues experimentally determined to be disordered which are predicted as disordered and true negatives are residues experimentally determined to be ordered and correctly predicted as ordered. False positives and false negatives are predictions which do not correspond to the experimentally determined state. Here, positive refers to disorder and so a false positive would be a residue incorrectly predicted to be disordered and a false negative would be a residue incorrectly predicted to be ordered.

The principle means used to evaluate the performance of our predictor are the area under the ROC curve (AUC) and the balanced accuracy (ACC). The ROC curve is a plot of sensitivity (i.e., SENS = TP ÷ (TP + FN)) against the false positive rate (i.e., FP ÷ (TN + FP)) across a variety of thresholds [40]. By calculating the area under the ROC curve it is possible to measure the general performance of a classifier irrespective of the decision threshold. The balanced accuracy is the simple average of the sensitivity and specificity (i.e., SPEC = TN ÷ (TN + FP)) using a decision threshold of 0.5. This evaluation metric is preferred over the accuracy given the disproportionate number of ordered residues compared to disordered residues in most datasets. In this setting, a naive classifier which classified all residues as ordered would have a very high accuracy but be useless for the task at hand. The same naive classifier would have a balanced accuracy of around 50%. In addition to the sensitivity, specificity, AUC and ACC, we also calculated a score (i.e., Sw = SENS + SPEC – 1) and the F-measure. All of these measures have been used extensively in the evaluation of other disorder predictors and in recent CASP assessments [1,14,19,22,41]. The significance of balanced accuracy, sensitivity, specificity, F-measure and Sw was obtained by approximating the standard error (SE) for each value. It was accomplished by a bootstrapping procedure in which 80% of the predicted residues where sampled 1000 times. More specifically, for a particular performance measure Θ, SE(Θ) = √(∑(Θ_i_ – Θ)^2^/1000) where Θ_i_ is the value of the measure calculated on the i^th^ sample.

### Methods used for comparison

In this study we compared our methods DNdisorder and PreDNdisorder against several predictors. Included in this comparison were several disorder predictors which are available publicly as servers or downloadable executables and several which participated in the CASP9 and CASP10 experiments. When selecting predictors from the CASP experiments, we included only those methods which performed particularly well in terms of ACC or AUC as determined by the official CASP9 assessment [1] or our in-house evaluation pipeline when applied to the CASP10 targets. Publicly available predictors used in our assessment included IUpred [11,12], ESpritz [14], PreDisorder [21] and CSpritz [42]. To generate disorder predictions, CSpritz was used as a web service while IUpred, ESpritz and PreDisorder were downloaded and run locally. For CASP participants, we downloaded disorder predictions from the official CASP website [23]. Note that when calculating the performance measures, the decision threshold was set to 0.5 for all methods (i.e., the same value used in the official CASP assessments) with the exception of ESpritz (when run locally) and CSpritz. In these two cases, we used decision thresholds of 0.0634 and 0.1225 respectively based on the accompanying documentation or output of these tools. One final caveat is that for the downloadable version of ESpritz (denoted in the results by Espritz_nopsi_X), we only report the results on predictions made by running ESpritz when trained X-ray structures and without profile information.

## Results and discussion

With nearly 60 disorder prediction methods and not all of them freely available, thoroughly benchmarking a new approach is a challenge. The situation is further exacerbated by different evaluation sets and metrics. As a basis for our analysis and comparison among disorder predictors, we used the Critical Assessment of Techniques for Protein Structure Prediction (CASP) experiment. This is a bi-annual, international experiment of various protein structure prediction methods including disordered regions. Over a period of approximately three months, protein sequences were released to the community and disorder predictions sent back to the prediction center. In CASP10, both DNdisorder (participating as MULTICOM-NOVEL) and preDNdisorder (participating as MULTICOM-CONSTRUCT) submitted disorder predictions to the prediction center along with approximately 26 other methods. In addition to the CASP10, we also benchmarked our novel approach against several disorder predictors on the CASP9 dataset. The comparison was made using evaluation metrics consistent with the literature and official CASP assessments [1,19,43,44].

We will also mention that we examined the pair wise sequence similarity between our training dataset DISORDER723 and the CASP9 and CASP10 datasets using NEEDLE [45]. We found that 8 of the CASP9 and 5 of the CASP10 protein targets had sequence similarities between 40-60% with a protein in the training set. The remaining CASP targets had sequence similarities less than 40% to proteins in the training set. To determine the impact of these relatively similar sequences, we evaluated DNdisorder on subsets of the CASP9 and CASP10 datasets with sequence similarity to the training data of less than or equal to 40%. There was no significant difference in terms of the ACC or AUC on the subsets compared to an evaluation over the full CASP datasets (data not shown). As the inclusion of the these 13 targets did not affect or enhance the performance of our methods, we used the performance of DNdisorder and PreDNdisorder on the full CASP9 and CASP10 datasets in our benchmark.

Tables 1 and 2 report the results of our methods on the CASP9 and CASP10 datasets. Both DNdisorder and PreDNdisorder compete competitively against state-of-the-art disorder predictors, particularly in terms of ACC. With respect to AUC on the CASP10 dataset, there are few methods such as PrDOS-CNF and biomine_dr which set themselves apart from the others while most of the predictors including PreDNdisorder and DNdisorder fall in the range 0.84-0.87. When ranking by ACC on the CASP10 dataset, both of our methods performed favourably with values in the range of 0.75-0.76, slightly behind the top performing method with an ACC value of 0.77.

**Table 1:** Performance on the CASP9 dataset

**Predictor**	**ACC**		**Sensitivity**		**Specificity**		**F-measure**		**Sw**		**AUC**	
	**Value**	**±SE**	**Value**	**±SE**	**Value**	**±SE**	**Value**	**±SE**	**Value**	**±SE**	**Value**	**±SE**
cspritz_server	76.00	0.54	66.21	1.0	85.79	0.36	43.46	0.89	52.00	1.1	0.8397	0.005
PRDOS2(291)	75.40	0.61	60.78	1.3	90.03	0.35	47.13	0.88	50.80	1.2	0.8544	0.005
espritz_nopsi_X	74.89	1.2	61.85	1.7	87.93	0.66	44.26	1.9	49.77	2.3	0.8301	0.005
DNdisorder	74.80	0.56	59.70	1.1	89.89	0.21	46.24	0.87	49.59	1.1	0.8299	0.005
PreDNdisorder	74.39	0.58	57.89	1.2	90.90	0.21	46.97	0.90	48.80	1.2	0.8396	0.005
PreDisorder	73.48	0.60	65.47	1.1	81.49	0.64	37.48	0.95	46.96	1.2	0.8136	0.005
biomine_dr_pdb (351)	74.12	1.1	59.45	1.6	88.49	0.67	43.94	1.9	48.23	2.2	0.8205	0.005
Multicom(490)	68.9	0.59	41.34	1.1	95.86	0.23	45.92	1.1	37.8	1.2	0.8550	0.005
DisoPred3C(15)	67.05	1.0	34.90	2.0	99.2	0.07	48.96	2.0	34.11	2.1	0.8539	0.005
iupred_short	63.36	0.69	32.06	0.14	94.67	0.17	34.84	1.3	26.73	1.4	0.6489	0.006

Results of a benchmark of DNdisorder, PreDNdisorder and a number of other disorder predictors. The evaluation was performed on 117 CASP9 targets consisting of 23656 ordered residues and 2427 disordered residues. The standard error (SE) is shown for each performance measure. Not included in this is assessment are 252 residues which were marked as ‘X’ by the CASP assessors. This table also contains four of the top performing methods from CASP9 according to the official CASP9 assessment. The predictions for these methods were downloaded from the official CASP website and the group number for these methods is provided in parenthesis. All values except AUC have been scaled by a factor of 100.

**Table 2:** Performance on the CASP10 dataset

**Predictor**	**ACC**		**Sensitivity**		**Specificity**		**F-measure**		**Sw**		**AUC**	
	**Value**	**±SE**	**Value**	**±SE**	**Value**	**±SE**	**Value**	**±SE**	**Value**	**±SE**	**Value**	**±SE**
metaprdos2(340)	77.06	0.92	64.73	1.4	89.40	0.98	41.24	2.9	54.12	1.8	0.8727	0.006
PreDisorder(125)	76.86	0.67	67.19	1.7	86.34	0.94	37.50	1.5	53.73	1.3	0.8394	0.006
POODLE(216)	76.84	0.78	62.74	1.6	90.94	0.26	43.06	1.0	53.68	1.6	0.8663	0.006
PreDNdisorder	76.55	0.75	61.74	1.8	91.36	0.61	43.42	1.5	53.10	1.5	0.8642	0.006
ZHOU-SPARKS-X (413)	75.68	0.76	64.81	1.4	86.55	0.96	36.43	1.9	51.36	1.5	0.8588	0.006
DNdisorder(424)	75.19	0.71	61.92	1.4	88.46	0.29	38.02	1.1	50.39	1.4	0.8480	0.006
CSpritz(484)	75.13	1.4	66.31	1.3	83.94	2.4	33.64	3.7	50.25	2.9	0.8215	0.007
Espritz(380)	73.16	1.6	59.24	1.4	87.08	2.6	34.58	4.7	46.31	3.2	0.8457	0.006
espritz_nopsi_X	71.98	0.97	53.10	1.5	90.87	0.77	37.56	2.4	43.97	2.0	0.8145	0.007
PrDOS-CNF(369)	70.35	0.88	41.95	1.8	98,74	0.14	52.5	1.4	40.70	1.8	0.8956	0.005
biomine_dr_mixed (478)	69.17	0.68	39.95	1.4	98.40	0.11	49.10	1.3	38.34	1.4	0.8844	0.006
biomine_dr_pdb_c (288)	67.81	1.2	36.88	2.6	98.74	0.15	47.65	2.1	35.62	2.5	0.8815	0.006
iupred_short	63.26	0.70	30.68	1.5	95.84	0.25	32.34	1.2	26.52	1.4	0.6642	0.007

Results of a benchmark of DNdisorder, PreDisorder, PreDNdisorder and a number of other disorder predictors. The evaluation was performed on 95 CASP10 targets consisting of 22673 ordered residues and 1597 disordered residues. The standard error (SE) is shown for each performance measure. Not included in this assessment are 1186 residues which were marked as ‘X’ by the CASP assessors. This table also includes a number of methods which participated in the CASP10 experiment. The predictions for these methods were downloaded from the official CASP website and the group number for these methods is provided in parenthesis. All values except AUC have been scaled by a factor of 100.

**Figure 4:**
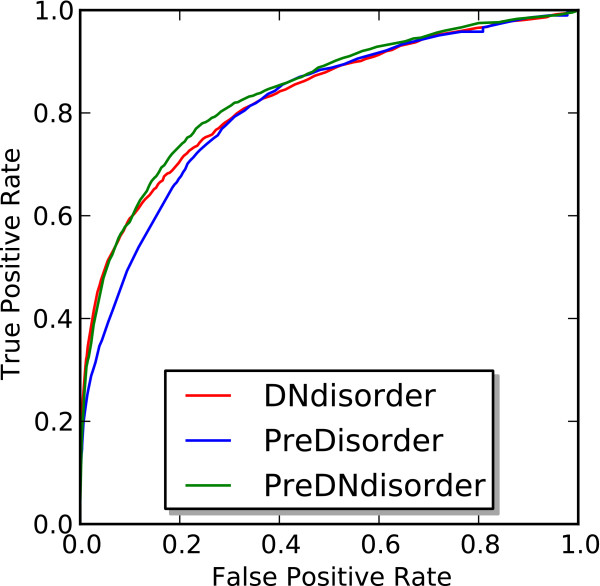
ROC curve of DNdisorder, PreDisorder and PreDNdisorder on CASP9 dataset.

**Figure 5:**
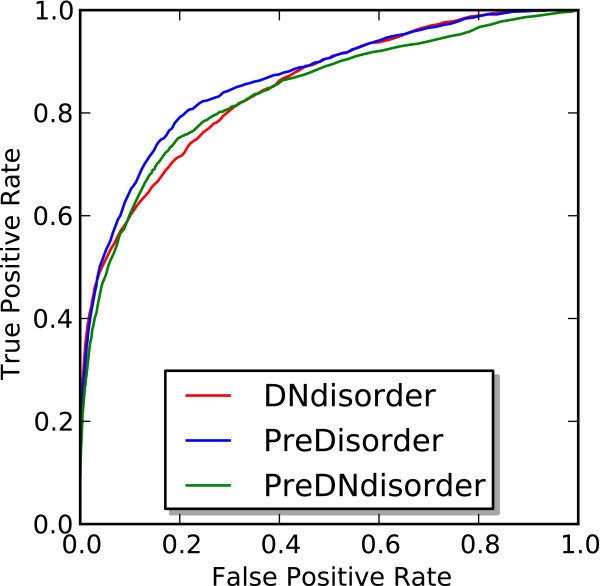
ROC curve of DNdisorder, PreDisorder and PreDNdisorder on CASP10 dataset.

On both the CASP9 and CASP10 evaluation sets, DNdisorder performed competitively against PreDisorder, a state-of-the-art disorder predictor as assessed in both CASP8 and CASP9 [1,19,22]. On the CASP10 evaluation set, our meta method PreDNdisorder slightly outperformed PreDisorder and our novel method DNdisorder and showed a modest improvement in AUC. This indicates that both PreDisorder and our novel approach are complementary in some respects as the combination of their respective predictions leads to a performance boost. To further investigate this point, we calculated the Pearson correlation coefficient between the scores assigned to disorder predictions by both PreDisorder and DNdisorder on the CASP9 dataset and was found to be 0.75. The additional time and complexity in running both methods and combining the results is negligible. We also generated ROC curves for DNdisorder, PreDisorder and PreDNdisorder for the CASP9 and CASP10 datasets and these are illustrated in Figures 4 and 5.

In addition to the evaluation on the CASP9 and CASP10 datasets we performed a 10 fold cross validation test on the DISORDER723 dataset. This is to say we divided the dataset up into 10 folds, all containing roughly the same number of proteins. Then 9 of the folds were used for training ensembles of boosted DN disorder predictors and then used to predict disorder of the proteins in the remaining fold. The average ACC of our approach DNdisorder was 0.82 and the AUC 0.90. Table 3 shows the results of all the performance measures for this 10 fold cross validation test.

**Table 3:** Performance of DNdisorder in a 10 fold cross validation test

**Predictor**	**ACC**		**Sensitivity**		**Specificity**		**F-measure**		**Sw**		**AUC**	
	**Value**	**±SE**	**Value**	**±SE**	**Value**	**±SE**	**Value**	**±SE**	**Value**	**±SE**	**Value**	**±SE**
DNdisorder	82.21	0.49	74.60	1.1	89.84	0.18	46.34	4.5	64.43	0.98	0.8995	0.002

Results of a 10 fold cross validation assessment of DNdisorder on the DISORDER723 dataset. The standard error (SE) is shown for each performance measure.

### Benefits of boosting

**Figure 6:**
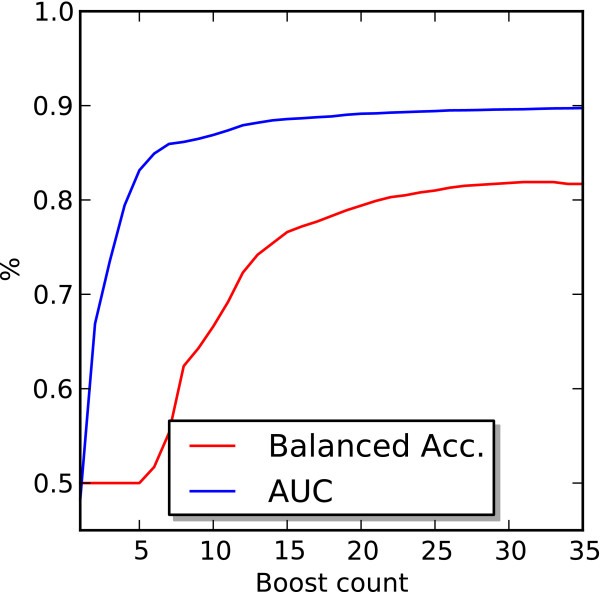
**Performance of a boosted ensemble.** To determine the effectiveness of boosting we evaluated the performance of an ensemble as a function of the number of rounds of boosting. The figure displays the balanced accuracy and area under the ROC curve for the DISORDER723 dataset. The predictions were made using a 10 fold cross validation procedure with input windows 30 residues long and a target window of 3 residues.

To determine the effect of boosting we evaluated the performance of the method as a function of the number of rounds of boosting. Figure 6 shows the ACC and AUC for an ensemble of DN predictors with an input window of 30 residues and a target window 3 residues in length. There is a clear improvement in performance with the AUC starting near .50 and quickly rising to around .86 and finally reaching near .90 after 35 rounds. The average balanced accuracy also showed a steady improvement reaching .82 after 35 rounds of boosting.

### Limitations

DNdisorder, as well as PreDisorder and PreDNdisorder, make use of information derived from PSI-BLAST. Using such information has been shown to result in a modest boost in performance but incurs a significant computational cost [14]. The web service we have developed for DNdisorder can process a protein of 250 residues in 10 to 20 minutes depending on server load. Consequently, our methods are not presently applicable to studies on a genomic scale. In the future we plan to develop predictors which do not depend on sequence profiles (i.e., information from PSI-BLAST), similar to the non PSI-BLAST implementations of Espritz which have been shown to be several orders of magnitude faster with only a marginal decrease in performance [14].

## Conclusions

In conclusion we have implemented a new framework for the prediction of protein disordered regions from sequence based on boosted ensembles of deep networks. In an evaluation with other state-of-the-art disorder predictor, our method DNdisorder performed competitively, indicating that this approach is capable of state-of-the-art performance. DNdisorder is available as a webservice at http://iris.rnet.missouri.edu/dndisorder/.

## Competing interests

The authors declare that they have no competing interest.

## Authors’ contributions

JE implemented the algorithms and carried out the experiments. JE and JC analyzed the data, wrote and edited the manuscript and approved it. Both authors read and approved the final manuscript.
